# m7G RNA methylation in cancer: Effect, mechanism and clinical application

**DOI:** 10.1002/ctm2.70521

**Published:** 2025-11-09

**Authors:** PengYuan Dang, KaiBo Li, ZhenQiang Sun

**Affiliations:** ^1^ Department of Colorectal Surgery The First Affiliated Hospital of Zhengzhou University Zhengzhou Henan China; ^2^ Henan Institute of Interconnected Intelligent Health Management The First Affiliated Hospital of Zhengzhou University Zhengzhou Henan China

**Keywords:** cancer, m7G, RNA methylation

## Abstract

**Background:**

RNA methylation has emerged as a pivotal layer of post‐transcriptional regulation that shapes the biological behavior of cancer cells. Among the diverse chemical modifications identified—such as N6‐methyladenosine (m6A), N1‐methyladenosine (m1A), 5‐methylcytosine (m5C), 7‐methylguanosine (m7G), 5‐hydroxymethylcytosine (5hmC), and 2′‐O‐dimethyladenosine (m6Am)—the m7G modification has recently garnered increasing attention. Mounting evidence indicates that m7G methylation plays an essential role in RNA metabolism and profoundly influences cancer initiation and progression.

**Main Topics:**

This Review synthesizes current advances in understanding the biological and clinical implications of m7G RNA methylation, with a particular focus on its key regulatory components, METTL1/WDR4 and eIF4E. We discuss how these enzymes and binding proteins orchestrate m7G deposition and recognition to modulate oncogenic processes, including cell growth, differentiation, metastasis, and therapeutic resistance. Furthermore, we highlight emerging evidence linking m7G‐related pathways to broader signaling networks that govern cancer plasticity and tumor microenvironment remodeling.

**Conclusions:**

m7G RNA methylation represents a rapidly evolving frontier in cancer epigenetics. The METTL1/WDR4 methyltransferase complex and eIF4E translation initiation factor have emerged as central nodes connecting RNA modification to oncogenic signaling. Targeting m7G‐dependent pathways holds considerable promise for the development of novel diagnostic biomarkers and therapeutic strategies. Continued exploration of this modification may ultimately expand the landscape of RNA‐based precision oncology.

**Key points:**

- m7G‐driven selective regulation exerts context‐dependent, two‐sided effects on tumour progression.
- m7G modulates therapeutic response, shaping chemosensitivity and resistance.
- m7G holds substantial clinical promise as a diagnostic/prognostic biomarker and a therapeutic target.

## Graphical abstract

m7G RNA methylation in cancer. m7G regulates various tumour‐related biological processes, including cell proliferation, metabolism, drug sensitivity, invasion, migration, immune evasion and anti‐tumour.

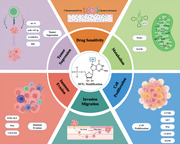

## 1 INTRODUCTION

Cancer progression is a complex, multi‐stage process governed by numerous regulatory factors. RNA methylation, as a specific class of RNA regulation, has been gaining attention in recent years for its role in biological functions.^1^, ^2^, ^3^ The m7G modification was initially recognized at the 5′ cap end of mRNA, which is also the most abundant modification here,^4^ and was immediately followed by the discovery of m7G modifications in prokaryotic, eukaryotic and archaeal tRNAs. The m7G modification of tRNA is mediated by the Trm8p/Trm82p complex in yeast and its human homolog METTL1/WDR4 (methyltransferase‐like 1/WD repeat structural domain 4), and has been linked to the onset and progression of several mammalian disorders.^5^, ^6^, ^7^, ^8^ With ongoing methodological advances in epigenetics and the maturation of computational RNA epigenetics pipelines, m7G methylation has been increasingly delineated across mRNA, miRNA and rRNA.^9^, ^10^ By governing essential processes in RNA metabolism, including pri‐miRNA maturation, mRNA translation, stability and export from the nucleus, they exert significant control over the onset and development of various cancers.^11^, ^12^ In addition, we predict its prospective and clinical application in targeted cancer therapy.

### 1.1 Biological properties of RNA methylation modifications in cancer

Biological macromolecules include DNA, RNA, proteins, sugars and lipids, all of which can be chemically modified to efficiently regulate their functions. Among these epigenetic modifications, Extensive investigations have been conducted on DNA and histone modifications. Recently, RNA modifications have gradually become a hot topic of interest.^13^, ^14^, ^15^ More than 170 chemical modifications have been discovered on RNA, with RNA methylation emerging as one of the most common and functionally diverse.^2^, ^3^, ^16^, ^17^ These RNA modifications are evolutionarily conserved and occur widely in both prokaryotic and eukaryotic systems. Found in multiple RNA species, including mRNA, tRNA, rRNA, snRNA and snoRNA, they play indispensable roles in the post‐transcriptional control of gene expression, governing RNA stability, splicing, processing and translational efficiency (TE), thereby ensuring proper cellular homeostasis and adaptive responses.^3^, ^18^ In particular, tRNA‐centred remodelling of translation has emerged as a key conduit linking tRNA‐modification status to proteome composition and oncogenic phenotypes.^19^, ^20^

With advances in liquid chromatography–tandem mass spectrometry (LC–MS/MS) and high‐resolution mapping, increasingly detailed profiles of RNA methylation have clarified their roles across transcription, translation, splicing and RNA stability. Beyond LC–MS/MS, methodologies for site‐resolved detection of m7G are advancing rapidly: base‐resolution mapping of internal m7G is now enabled by borohydride‐reduction mutational profiling,^21^ aniline‐assisted cleavage^22^, ^23^ and tRNA‐focused reduction‐and‐cleavage,^24^ while m7G‐quant‐seq introduces calibrated stoichiometry estimates for tRNA/rRNA sites.^25^ Complementary resources such as m7GHub v2.0 curate cross‐species m7G sites and annotations.^26^ Accumulating studies have revealed that dynamic RNA methylation modifications are intricately involved in cell survival, proliferation, invasion, stress, epithelial‐mesenchymal transition (EMT), tumour microenvironmental adaptation, immune evasion and chemoresistance.^27^, ^28^, ^29^ These modifications also intersect tumour metabolism and the tumour microenvironment. Studies show RNA‐methylation programmes can rewire lipid metabolism in nasopharyngeal carcinoma and engage Wnt/TME signalling axes to shape malignant progression.^30^, ^31^ Moreover, RNA methylation modulates anti‐tumour immunity by affecting T‐cell differentiation, dendritic‐cell maturation and Treg status, with important implications for immune activation and immune evasion.^2^, ^3^, ^17^, ^32^, ^33^ Accumulating evidence thus supports dysregulated RNA methylation as a key driver of tumour progression,^34^, ^35^, ^36^, ^37^ while related RNA technologies highlight translational opportunities in oncology immunotherapy.^38^

### 1.2 Core molecules of m7G RNA modification

**FIGURE 1:**
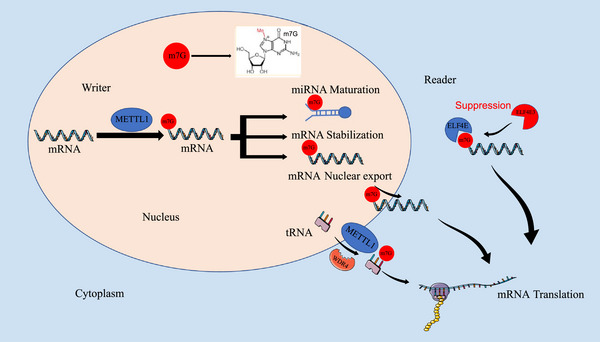
Core molecules of m7G methylation modification, including the methylation molecule METTL1 and the recognition molecule eIF4E. METTL1 is the ‘writer’ of m7G, which promotes pri‐miRNA processing and maturation, enhancing mRNA stability, translation and nuclear export. In mammals, tRNA m7G modification is mediated by the METTL1/WDR4 complex, which regulates the translation of oncogenic mRNAs. eIF4E is the ‘reader’ of m7G, which binds to the 7‐methylguanosine (m7G) cap on mRNAs to participate in the translation process.

The core molecules of m7G RNA methylation include the methylation molecule METTL1 and the recognition molecule eIF4E. METTL1 is the most important catalase in mediating m7G methylation.^5^ It belongs to the superfamily of S‐adenosylmethionine‐dependent enzymes, which is essential for mediating diverse biological methylation reactions.^37^ METTL1 promotes gene expression, RNA metabolism and maintenance of protein stability in humans.^37^, ^39^ The m7G modification is essential not only for the formation of the 5′ cap structure of mRNA but also occurs at internal sites along the mRNA molecule. METTL1 is the m7G mRNA modification ‘writer’ (Figure 1). It orchestrates multiple post‐transcriptional processes, such as mRNA processing, translation, stability and export from the nucleus.^36^, ^37^, ^40^, ^41^, ^42^, ^43^, ^44^, ^45^ In lung cancer (LC), METTL1 disrupts the stability of its G‐quadruplex structure by methylating let‐7e pri‐miRNA, thereby enhancing miRNA processing and maturation, which in turn reduces the migratory capacity of cells.^5^, ^11^ Similarly, METTL1 regulates the let‐7e miRNA/HMGA2 axis through m7G methylation modification to inhibit colon cancer (CC) progression.^46^ It was found that METTL1 can regulate m7G tRNA modifications and expression across multiple cancer types.^47^, ^48^ This modification exerts selective control over the selective translation of oncogenic mRNAs governed by a codon frequency–dependent mechanism, primarily orchestrated by the METTL1/WDR4 complex, thereby influencing tumour growth and malignancy. Among them, METTL1 acts as a methylation molecule with catalytic activity and WDR4 maintains the protein abundance of METTL1 and is deeply involved in the regulation of stabilizing the conformation of the METTL1/WDR4. Studies showed that WDR4 can also bind directly to RNA, resulting in stronger interactions.^5^, ^6^, ^7^ In addition, c‐MYC directly upregulates WDR4 at the transcriptional level, promoting increased mRNA translation and stabilization.^49^

eIF4E, a key eukaryotic translation initiation factor, frequently acts as the ‘reader’ molecule that identifies and binds m7G‐modified RNA. It is a strong oncogene that is widely present in many human cancers. eIF4E is part of a gene family comprising three members that share conserved structural domains and related biological functions: eIF4E1, eIF4E2 and eIF4E3. They can bind the m7G cap on mRNA and are also known as cap‐binding proteins.^50^ They binds the m7G cap between two aromatic residues, a configuration that enables specific molecular recognition and binding essential for cap‐dependent translation initiation.^51^ In addition, a more established research model shows that eIF4E3 competes with eIF4E for the same transcriptional pool to inhibit the oncogenic capacity of eIF4E to drive tumour development.^52^ eIF4E localizes to both the nucleus and cytoplasm, where it performs distinct regulatory roles. Within the nucleus, eIF4E associates with the m7G cap of mRNAs to promote their export, while in the cytoplasm, it mediates mRNA recruitment to ribosomes during translation initiation and enhances protein synthesis efficiency.^53^, ^54^, ^55^, ^56^

### 1.3 Mechanisms and biological functions of m7G RNA methylation in cancer regulation

**FIGURE 2:**
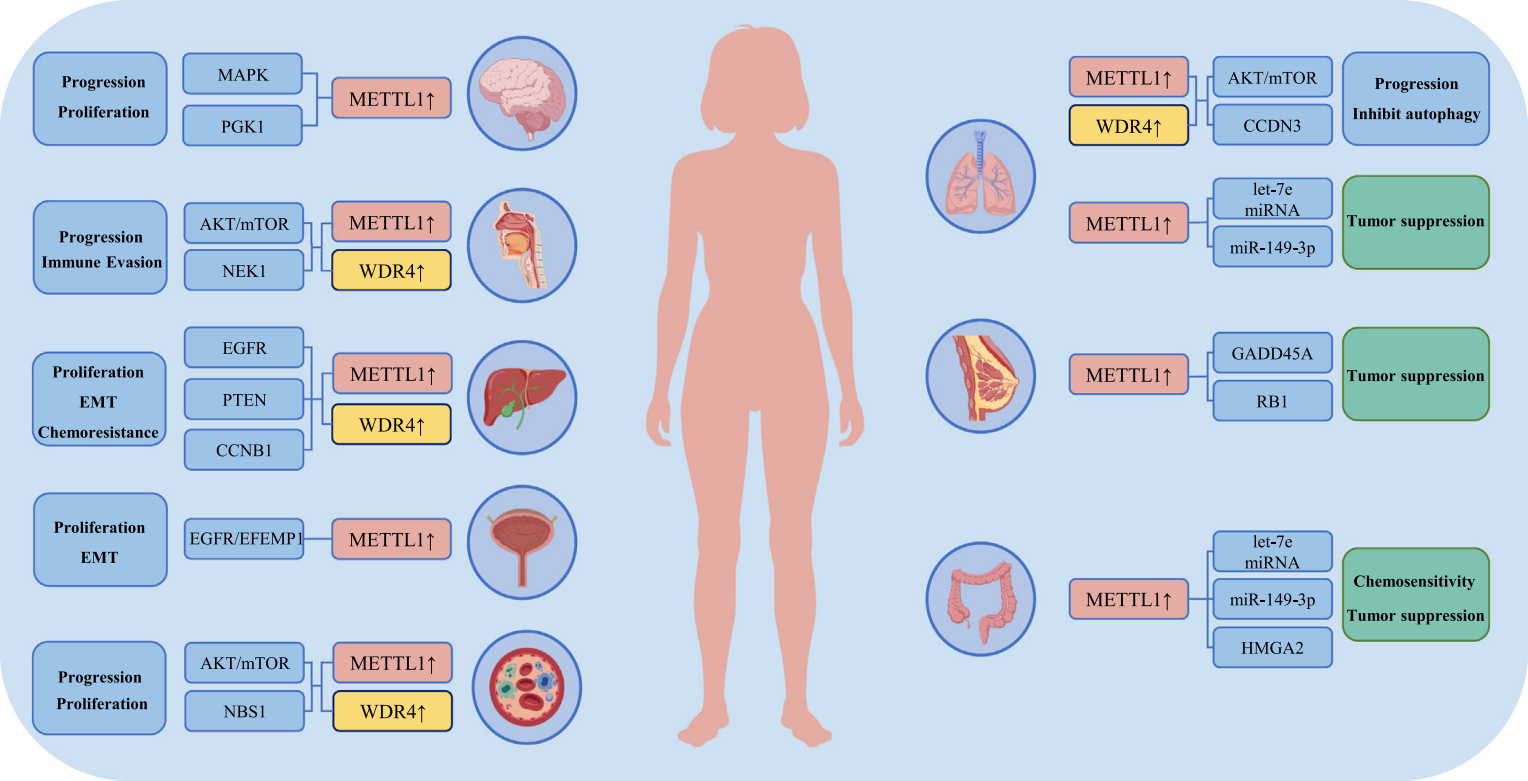
m7G RNA methylation regulates specific mechanisms and biological functions of cancer. m7G modifications activate oncogenic signalling pathways and regulate the expression of cancer‐associated genes through various mechanisms. Then, they cause cell proliferation, growth, invasion, metastasis, apoptosis, epithelial‐mesenchymal transition (EMT), chemoresistance, immune evasion, which in turn affect cancer development and progression.

m7G RNA methylation has essential biological regulatory functions in the development of cancer. In this section, we highlight the specific mechanisms and functions by which m7G RNA methylation modulates RNA metabolism and contributes to tumour development (Figure 2).

In primary hepatocellular carcinoma (HCC), m7G RNA methylation modulates oncogenic mRNA translation in a decoding codon–dependent fashion. METTL1 knockdown diminishes TE, thereby lowering the expression levels of key oncogenic proteins, including epidermal growth factor receptor (EGFR), VEGFA and Cyclin A2. Then, downregulation of AKT and MAPK activities, signals downstream of VEGFA/VEGFR1 signalling pathway, restrained both the proliferative capacity and metastatic behaviour of HCC cells. knockdown of METTL1 inhibited Cyclin A2 translation, causing G2/M cell cycle arrest. HCC cell culture showed that METTL1 overexpression promoted the growth and migration of HCC cells. In addition, METTL1 overexpression can regulate the phosphatase and tensin homologue (PTEN) signalling pathways that are absent on chromosome 10 to promote HCC cell proliferation and migration.^47^, ^49^, ^57^, ^58^ MYC is an important oncogenic transcription factor that promotes cell cycle progression.^59^, ^60^ Studies have confirmed that MYC activates WDR4 transcription by binding to the WDR4 promoter region.^48^ WDR4 enhances eIF2A binding to mRNA, promoting the translation and stability of CCNB1, consequently leading to activation of the PI3K/AKT signalling pathway and ubiquitination of P53 in HCC. Similarly, in intrahepatic cholangiocarcinoma (ICC), METTL1‐ driven m7G tRNA modification drives the translational efficiency of oncogenic transcripts,^61^, ^62^ including EGFR signalling pathways and cell cycle genes, thereby contributing to cancer progression.

As previously described, METTL1 inhibits LC progression by modulating the let‐7e miRNA/HMGA2 axis. m7G modification promotes maturation of miRNA processing to inhibit LC cells migration.^9^ Similar findings were found in colon cancer, in which METTL1 overexpression inhibited proliferation and migration of CC cells. In addition, METTL1 can regulate apoptosis of CC cells. By targeting HMGA2, it inhibits proliferation and migration, ultimately restraining cancer progression. miR‐149‐3p has been identified as a tumour‐suppressive miRNA in LC, and METTL1 promotes its expression in an m7G‐dependent fashion, contributing to the inhibition of tumour progression. Consistently, elevated expression of both METTL1 and WDR4 have been reported in LC tissues.^63^ Silencing METTL1 markedly attenuated the invasiveness of A549 and H1299 cells, and depletion of WDR4 led to reduced cellular proliferation. Furthermore, reintroduction of CCND3, a downstream effector of METTL1, partially restored the proliferation and metastatic capacity of METTL1‐deficient A549 lung cancer cells. These results confirm that METTL1 and WDR4 act as oncogenic drivers in lung cancer by m7G tRNA modifications to regulate mRNA translation. Consistent with its oncogenic role, METTL1 was reported to facilitate A549 cell proliferation and induce autophagy through upregulation of the AKT/ mechanistic target of rapamycin complex 1 (mTORC1) axis, highlighting its contribution to tumour growth and metabolic adaptation.^64^

Beyond these entities, emerging evidence in breast cancer (BC) reveals important roles of the m7G axis.^65^ In defined molecular settings, METTL1‐mediated tRNA m7G induces translational dysfunction and enforces cell‐cycle arrest, thereby limiting tumourigenesis.^66^ Complementarily, integrative models that combine m7G regulators and their related genes with tumour‐microenvironment features have been proposed as prognostic and therapeutic biomarker signatures in BC,^67^ while multi‐omic profiling further underscores the clinical relevance of m7G regulators in this disease.^65^ In addition, crosstalk between m7G‐related pathways and the miRNA machinery has been noted, for example, aberrant expression of the m7G‐related gene AGO2 modulates BC cell invasion by regulating LASP1 and let‐7a levels.^68^

In oral squamous cell carcinoma (OSCC), evidence indicates that METTL1/WDR4‐driven tRNA m7G enhances selective translation of growth and EMT programmes, thereby promoting tumour progression.^69^ Growing evidence supports the notion that METTL1 catalyses the m7G methylation of NEK1 mRNA, causing increased transcript stability and elevated NEK1 protein production. This regulatory mechanism promotes tumour cell proliferation and underscores the oncogenic potential of METTL1‐mediated m7G modification.^70^ These findings nominate METTL1 as a tractable node in OSCC, operating through both tRNA‐dependent translational control and mRNA‐centred regulation.

METTL1 is highly expressed in bladder cancer (BCa).^48^ m7G tRNA modification mediated by METTL1 promotes the expression of EGFR/EFEMP1 protein, which intercalates EGFR with its ligand EFEMP1 (fibronectin‐like extracellular matrix protein 1 containing EGF). Then, subsequent activation of the PI3K/AKT signalling cascade stimulates cancer cell proliferation and motility. Additionally, METTL1‐mediated m7G methylation modulates the abundance and functionality of specific tRNAs, thereby selectively improving the translation of transcripts enriched in m7G‐favored codons and facilitating oncogenic protein synthesis. This regulation drives the occurrence and malignant progression of BCa by facilitating the selective translation of key oncogenic proteins.

METTL1 overexpression is significantly linked to glioma development and aggressiveness. Studies have indicated that METTL1‐mediated m7G may activate the MAPK pathway to promote glioma growth and proliferation.^37^ More recent functional evidence indicates that METTL1 drives glioma development through m7G‐dependent modification and stabilization of the glycolytic enzyme PGK1 mRNA, which enhances glycolytic activity, promotes cellular proliferation and accelerates tumour growth in vivo.^71^ In line with this, a comprehensive epitranscriptomic overview in glioma further contextualizes m7G regulators—including METTL1/WDR4 and cap‐dependent readers, within signalling networks and clinical relevance.^72^

eIF4E exerts its oncogenic effects primarily through its ability to bind the m7G cap at the 5′ end of mRNAs, which enhances both translation initiation and nuclear export. Overexpression of eIF4E has been linked to the upregulation of multiple pro‐tumourigenic regulators, such as ornithine decarboxylase, fibroblast growth factor and vascular endothelial growth factor (VEGF), all of which contribute to tumourigenesis. Additionally, eIF4E facilitates AKT activation by promoting the expression of NBS1, a protein that interacts with PI3K, thereby amplifying downstream signalling pathways. In acute myeloid leukaemia (AML), this interaction between eIF4E and NBS1 plays a pivotal role in activating AKT, which supports cancer cell survival and proliferation.^73^ Activation of this signalling pathway stimulates the nuclear export and translational efficiency of selective mRNA transcripts, contributing to increased cancer cell growth and proliferation. Despite these findings, the precise molecular mechanisms and context‐specific roles of this pathway in cancer development warrant deeper investigation.

### 1.4 Impact of m7G RNA methylation on drug sensitivity

Drug resistance is a pervasive obstacle in cancer therapy, often leading to treatment failure, tumour occurrence and poor patient outcomes. The emergence of drug resistance represents a critical factor underlying tumour recurrence and metastatic dissemination, posing a major obstacle to effective cancer therapy. The growing field of RNA epigenetics has revealed that m7G methylation represents a crucial epitranscriptomic modification governing cancer chemoresistance. It exerts its effects by modulating RNA stability, TE and global gene expression networks, thereby reshaping cellular responses to anticancer therapies (Table 1). METTL1 increases the chemosensitivity of CC cells to cisplatin by regulating the miR‐149‐3p/S100A4/p53 axis, thereby enhancing apoptosis and inhibiting tumour cell survival under chemotherapeutic stress.^74^ Similarly, in cervical cancer, METTL1 enhances the sensitivity of HeLa cells to 5‐fluorouracil (5‐FU) drugs.^75^ Recent study reported that WDR4 was significantly overexpressed and increased levels of m7G methylation in HCC. It enhances resistance to sorafenib by promoting EMT and translation of CCNB1.^49^ In parallel, METTL1‐catalyzed m7G modification of tRNA contributes to lenvatinib resistance in HCC by selectively elevating TE of genes within the EGFR signalling, thereby sustaining oncogenic signalling and chemoresistance.^76^ Consistent with these observations, in OSCC, METTL1‐driven tRNA m7G reprogrammes tumour metabolism and promotes acquired resistance to the multi‐target TKI anlotinib. Genetic or pharmacologic attenuation of METTL1 restores drug sensitivity in vitro and vivo, implicating an m7G‐translation‐metabolism axis as a tractable vulnerability.^77^ The eukaryotic translation initiation factor 4F (eIF4F) complex serves as a key mediator of cap‐dependent translation initiation. It consists of three core components: eIF4E, eIF4A, eIF4G and other initiation factors. Like eIF4E, the eIF4F complex specifically recognizes and attaches to the m7G cap structure at the 5′ end of mRNA, facilitating cap‐dependent translation initiation. Resistance to the eIF4F complex relies on mechanisms such as reactivation of MAPK signalling, phosphorylation of the eIF4E translational repressor 4EBP1 and degradation of BMF‐eIF4G.^78^, ^79^, ^80^, ^81^, ^82^, ^83^, ^84^, ^85^, ^86^, ^87^ Therapeutic strategies combining BRAF inhibitors with agents targeting eIF4F have demonstrated the ability to overcome most resistance pathways that emerge in cancers carrying the BRAF (V600) mutation such as melanoma, colon and thyroid cancers.

**TABLE 1:** m7G‐related drug sensitivity and resistance across different cancer types.

Cancer type	Drug	regulators	Mechanism	Function	PMID
Colon cancer	Cisplatin	METTL1	miR‐149‐3p/S100A4/p53 axis	Increases drug sensitivity	31866582
Cervical cancer	5‐FU	METTL1	Promotes tRNA translation	Increases drug sensitivity	25233213
Hepatocellular carcinoma	Sorafenib	WDR4	Promotes CCNB1 translation	Promotes drug resistance	34211143
Hepatocellular carcinoma	Lenvatinib	METTL1	Promotes EGFR pathway translation	Promotes drug resistance	36102722
Oral squamous cell carcinoma	Anlotinib	METTL1	Promotes oxidative phosphorylation	Promotes drug resistance	38280546
Acute myeloid leukaemia	Ribavirin	eIF4E	Enhances oncogenic mRNA translation	Promotes drug resistance	19433856, 37679400
Melanoma	Vemurafenib	eIF4F	Activates MAPK pathway	Promotes drug resistance	25079330, 22858545, 24265155

Abbreviation: EGFR, epidermal growth factor receptor.

### 1.5 Two‐sided characteristic of m7G RNA methylation in cancer regulation

**FIGURE 3:**
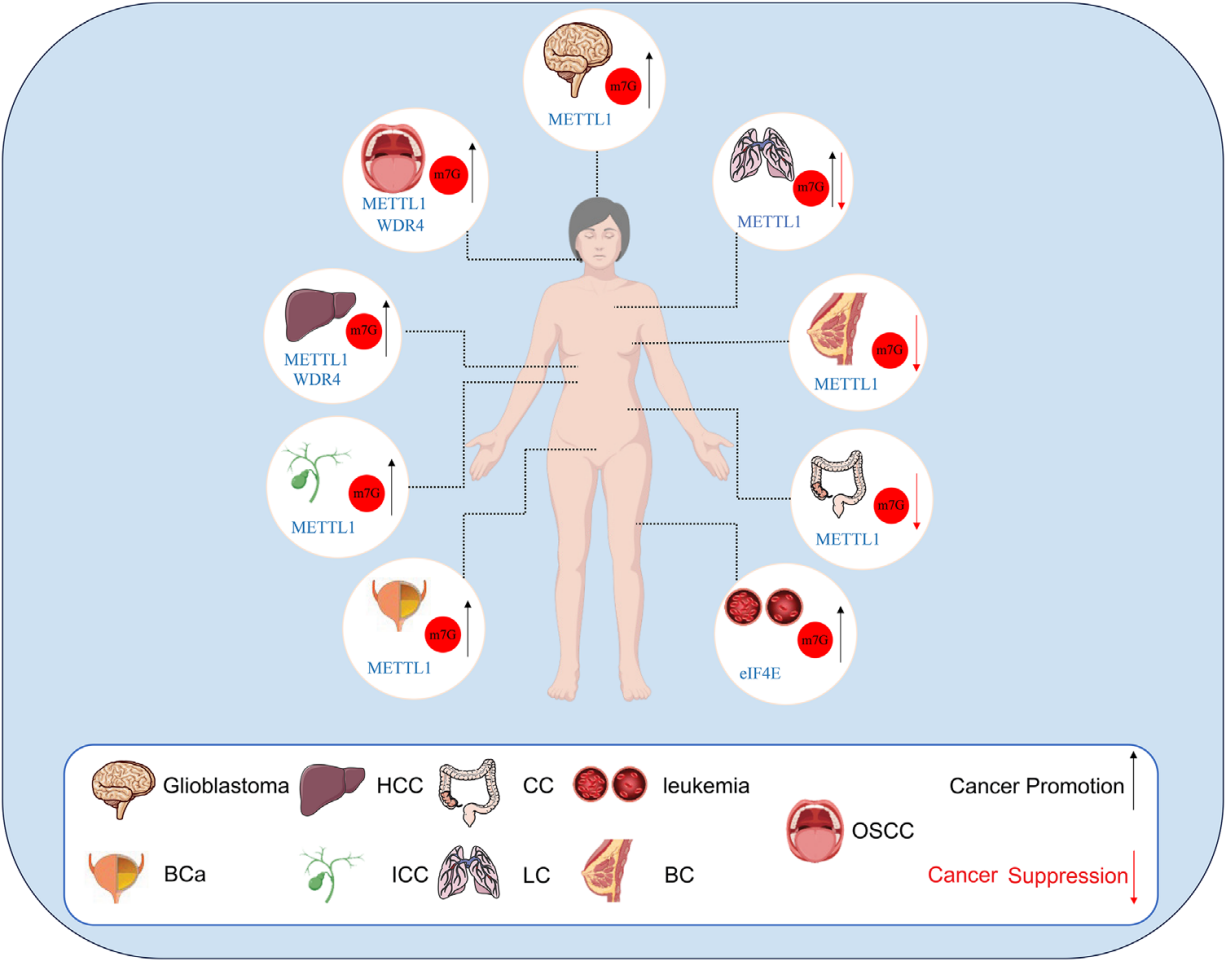
m7G methylation modifications are associated with the progression of a variety of malignancies, including hepatocellular carcinoma (HCC), intrahepatic cholangiocarcinoma (ICC), liquid chromatography (LC), colon cancer (CC), bladder cancer (BCa), breast cancer (BC), oral squamous cell carcinoma (OSCC), glioblastoma, leukaemia and others. m7G regulation in cancer is two‐sided, producing either pro‐tumour or tumour‐suppressive effects depending on context.

In contrast to its broadly oncogenic activity in HCC, ICC, BCa and glioma, accumulating evidence indicates that m7G/METTL1 can exert tumour‐suppressive effects in lung cancer, colon cancer, and, in defined contexts, breast cancer (Table 2 and Figure 3). In LC, METTL1‐dependent m7G enhances pri‐miRNA maturation, notably within the let‐7 family and the miR‐149‐3p pathway, which in turn represses HMGA2 and S100A4 and constrains migration and progression.^9^, ^74^ In CC, METTL1 overexpression has been associated with greater cisplatin sensitivity via the miR‐149‐3p axis, consistent with a tumour‐suppressive output of the m7G–miRNA programme in a therapeutic context.^74^ In BC, recent work likewise reports tumour‐suppressive facets of the m7G axis in specific molecular settings, supporting a context‐dependent restraint on tumour progression.^66^

**TABLE 2:** Two‐sided characteristic of m7G RNA methylation in cancer regulation.

Cancer type	Role of m7G	Regulators	Molecular axis	Function	PMID
HCC	Oncogenic	METTL1/WDR4	EGFR pathway, PTEN, CCNB1	Promotes proliferation, migration, EMT and drug resistance	31463732, 34898034, 34244479
ICC	Oncogenic	METTL1	EGFR pathway, CXCL8, Cell cycle	Promotes proliferation and tumour survival	34352206
Lung cancer	Oncogenic	METTL1/WDR4	CCDN3, AKT/mTOR pathway	Promotes proliferation, migration and invasion; Inhibits autophagy	34371184, 33692862
Lung cancer	Tumour suppressive	METTL1	let‐7e miRNA, miR‐149‐3p	Inhibits proliferation and invasion	31031083
Colon cancer	Tumour suppressive	METTL1	miR‐149‐3p, let‐7e miRNA, HMGA2	Inhibits proliferation, migration and increase drug sensitivity	31866582
Breast cancer	Tumour suppressive	METTL1	Cell cycle: GADD45A, RB1	Inhibit tumour occurrence and progression	38822363
OSCC	Oncogenic	METTL1/WDR4	PI3K/AKT/mTOR pathway, NEK1	Promotes progression and immune evasion	35179319, 40562282
Bladder cancer	Oncogenic	METTL1	EGFR/EFEMP1	Promotes EMT and proliferation	34936728
Glioma	Oncogenic	METTL1	MAPK pathway, PGK1	Promotes proliferation	34838021,40815965

Abbreviations: EGFR, epidermal growth factor receptor; EMT, epithelial‐mesenchymal transition; HCC, hepatocellular carcinoma; ICC, intrahepatic cholangiocarcinoma; OSCC, oral squamous cell carcinoma; PTEN, phosphatase and tensin homologue.

This suppression in LC/CC/BC, contrasted with oncogenesis in HCC, is unlikely to reflect sample heterogeneity alone; rather, it points to context‐dependent regulatory networks and tissue‐specific cofactors that steer METTL1‐coupled programmes toward miRNA maturation (suppressive) or toward selective translation (oncogenic). In HCC, oncogenic transcriptional states, exemplified by MYC‐driven WDR4 upregulation, favour tRNA‐m7G‐dependent, codon‐biased translation of cell‐cycle and survival mRNAs, thereby amplifying PI3K/AKT and MAPK signalling and promoting proliferation, metastasis and drug resistance. By contrast, in LC and CC, and in subsets of BC defined by receptor or signalling context, the prevailing RNA‐binding‐protein landscape (typified by the LIN28/hnRNP balance), together with tumour‐suppressive checkpoints and pathway tone (intact TP53/PTEN and restrained Wnt/β‐catenin) and a cap‐reader equilibrium that favours eIF4E3 over eIF4E1, biases METTL1 outputs towards miRNA‐centred repression rather than translational activation, yielding a tumour‐suppressive phenotype in LC and, under treatment conditions, chemosensitization in CC.^9^, ^52^, ^66^, ^74^

### 1.6 The impact of m7G methylation of different RNAs on cancer progression

**FIGURE 4:**
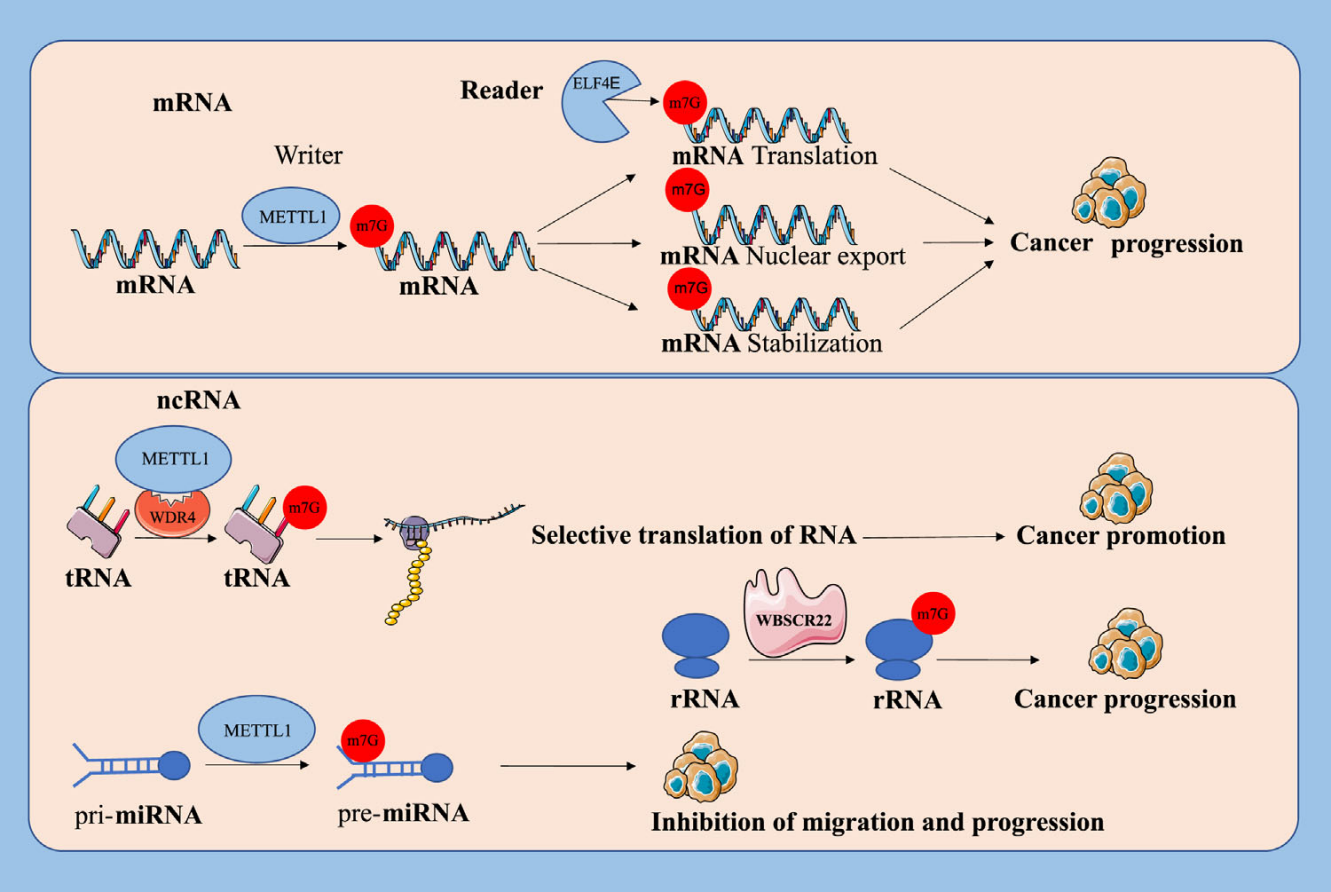
The m7G modification acts on coding and non‐coding RNA. m7G is located at or within the 5′ cap end of mRNA and is cross‐linked by the ‘author’ METTL1 and ‘reader’ eIF4E to stabilise the structure of mRNA, facilitate mRNA export and translation initiation. In tRNAs, METTL1‐WDR4‐mediated m7G modification lead to cancer promotion through selective translation. In miRNAs, METTL1‐mediated m7G modification inhibit cancer cell migration and progression through maturation of precursor miRNA processing. In rRNAs, m7G modification is mediated by WBSCR22 and is associated with cancer progression.

Accumulating evidence suggests that m7G RNA methylation is present in all types of RNA molecules (Figure 4).^8^, ^21^, ^40^, ^88^, ^89^ m7G RNA methylation modify both messenger RNA (mRNA), and non‐coding RNA (ncRNA), including miRNA precursors (pri‐miRNA), transfer RNA (tRNA) and ribosomal RNA (rRNA).

### 1.7 m7G in mRNA

m7G methylation was originally characterized as a fundamental component of the 5′ cap structure of mRNA. m7G capping modification facilitates the maturation of mRNA processing, maintains stability and promotes nuclear export.^40^, ^42^, ^43^, ^44^, ^45^, ^90^, ^91^ In AML, eIF4E controls the nuclear export and translation of selective mRNA through its specific recognition and binding of the m7G 5′ end cap structure, which in turn leads to cancer progression. Subsequently m7G methylation was also found within mammalian mRNA.^9^ METTL1‐WDR4 acts as an m7G methyltransferase complex of mRNA and mediates m7G methylation within mRNA. This m7G methylation affects the translation process and leads to the development of cancer.

In conclusion, the regulation of mRNA by m7G modifications has important implications for cancer progression. We believe that more mRNA regulatory mechanisms of m7G modifications will be discovered in the future.

### 1.8 The m7G of non‐coding RNA (ncRNA)

miRNAs constitute a group of small, highly conserved and abundantly expressed noncoding RNAs that exert post‐transcriptional control over expression, primarily by promoting the decay of target mRNAs or by blocking translation, thereby shaping diverse cellular processes and disease states. miRNA primary transcripts (pri‐miRNAs) undergo a series of processing to form precursor miRNAs (pre‐miRNAs) and mature miRNAs.^92^, ^93^, ^94^, ^95^ In recent years, m7G methylation has been detected in human miRNA precursors. METTL1 was shown to methylate let‐7e primary miRNA precursors in lung cancer cells, leading to the maturation of miRNA processing. This m7G methylation modification promotes LC development by stimulating cancer cell migration, invasion and overall tumour progression, highlighting its potential role as an oncogenic regulator. Similar findings in CC also argue for this phenomenon.

tRNA recognizes codons on mRNA and brings the corresponding amino acids, which are important components of protein translation. tRNA is most abundant in RNA modifications, and precise tRNA modifications maintain tRNA stability, promote codon recognition and protein synthesis.^75^, ^96^, ^97^, ^98^ m7G methylation is predominantly reported in the variable loop of tRNA. It contributes to tRNA stability and translational fidelity. Recent studies have uncovered that this modification exerts a pivotal influence on cancer progression by enhancing oncogenic translation and tumour cell adaptability.^99^, ^100^ For example, METTL1‐WDR4 complex‐mediated m7G tRNA methylation improves translational efficiency of cell cycle related genes and oncogenic mRNAs and has an impact on the activity of tumour progression pathway in HCC. Similarly, this selective mechanism of mRNA translation regulation has been extensively documented in diverse human cancers, such as ICC, LC, CC and BCa.

rRNA is indispensable for mRNA translation in eukaryotes, providing the structural framework and catalytic activity of the ribosome. It comprises four distinct components, called 5S, 5.8S, 18S and 25S/28S rRNAs. Various processes of ribosomal processing are potentially affected by chemical modifications.^101^, ^102^, ^103^, ^104^ Studies have confirmed that WBSCR22 is an RNA methyltransferase that mediates m7G methylation on rRNA.^105^, ^106^ WBSCR22 catalyses m7G methylation at nucleotide G1639 of 18S rRNA, which is crucial for proper processing of nuclear 18S rRNA precursors. Loss of WBSCR22 expression drives defective 18S rRNA maturation and abnormalities in ribosome biogenesis. The depletion of WBSCR22 has been associated with cancer progression and inflammatory responses. Another study demonstrated that 66 distinct oligonucleotide sequences within the primary structure of 18S rRNA isolated from Novikoff hepatoma cells contain m7G methylation modifications. This finding aligns with earlier reports and further supports the conserved and widespread occurrence of m7G methylation in ribosomal RNA from tumour cells.^107^

In summary, as the study of m7G modifications continues to progress, m7G modifications are widely present on ncRNAs and contribute critically to the multistep process of tumour initiation and subsequent progression across diverse cancers. In addition, the detection of m7G sites for various non‐coding RNAs such as circular RNA (CircRNA) and long‐stranded non‐coding RNA (lncRNA) will be gradually carried out.^108^

### 1.9 Upstream‐related regulators of m7G RNA methylation

Different variants of eIF4E regulate the m7G RNA methylation modification process. eIF4E is markedly overexpressed in various epithelial cancers, including OSCC, BC, CC and BCa. Its elevated expression is closely linked to enhanced tumour progression and aggressive clinical behaviour. Experimental studies have shown that downregulating eIF4E expression in cancer cells diminishes their proliferative and transformative properties, highlighting eIF4E pivotal role in oncogenesis. A recent study introduced MDA‐MB‐435 cell lines expressing different eIF4E variants, which provided insights into how variations in eIF4E function could influence cancer cell behavior.^109^ Cell lines overexpressing the eIF4E variant had impaired 5′ cap m7G binding function and showed a different morphology compared to control cell groups. eIF4E variants, which exhibit reduced affinity for the mRNA cap, significantly alter the phenotype of MDA‐MB‐435 cancer cells. This change disrupts the normal regulation of mRNA translation, resulting in altered cell proliferation rates and altered cellular behaviours that contribute to tumourigenesis. A decrease in eIF4E binding affinity for the mRNA cap leads to diminished translation of key growth‐regulatory proteins, ultimately leading to a shift in cellular proliferation dynamics. Thus, the m7G RNA methylation involved in eIF4E is regulated by the transcripts of different genes.

Promyelocytic leukaemia protein (PML) regulates cell growth and apoptosis in mammals. eIF4E overexpression causes oncogenic transformation, while PML overexpression inhibits oncogenic transformation. This altered physiological function is dependent on the expression of the oncogenic domain of PML. PML contains a cysteine‐rich RING domain that binds to the dorsal surface of eIF4E, thereby significantly diminishing its ability to recognize and bind the m7G cap structure at the 5′ end of mRNA.^110^, ^111^, ^112^, ^113^, ^114^, ^115^ In conclusion, the RING of PML acts as one of the upstream‐related regulators of m7G RNA methylation. Its oncogenic transformation is achieved through the active regulation of the transport function.^116^

### 1.10 The impact of m7G RNA methylation on various cancer progression

Primary liver cancer is among the most lethal malignancies, ranking fourth in global cancer mortality and posing a major public health burden worldwide. For advanced HCC, transarterial chemoembolization and targeted drug therapy are currently recommended. However, their treatment effect is unsatisfactory. The low survival rate and high recurrence rate have been an accepted challenge in the industry.^117^, ^118^, ^119^ As mentioned above,^47^ some researchers used patient samples, an in vitro culture model of HCC cells, and a METTL1 knockout mouse model to confirm the critical role of METTL1/WDR4‐mediated m7G tRNA modification in promoting HCC development. m7G tRNA modification promotes HCC progression in vivo and in vitro. In addition, METTL1 also facilitates HCC progression by suppressing PTEN expression, which in turn activates the AKT pathway.

ICC accounts for 10%–20% of all primary liver cancers. Even after surgical resection and aggressive chemotherapy, the 5‐year survival rate of ICC patients is only 5%–40%.^120^, ^121^ This poor prognosis is associated with elevated levels of METTL1/WDR4‐mediated m7G methylation. m7G tRNA methylation mediated by METTL1 promotes the development and progression of ICC by performing its key tumourigenic functions.

LC is the most prevalent malignancy, accounting for a substantial proportion of deaths among cancer patients each year. Evidences have suggested that dysregulated epigenetic modifications are key drivers of LC progression, influencing gene expression, tumour heterogeneity and therapeutic resistance.^122^, ^123^, ^124^ METTL1 is overexpressed in LC tissues and is strongly associated with poor prognosis. METTL1 and WDR4‐mediated m7G tRNA modifications exert oncogenic effects through selective translation. METTL1 also regulates MiR‐149‐3p axis through m7G methylation and let‐7e miRNA/HMGA2 axis to inhibit lung cancer cell proliferation and migration, causing LC progression.

CC is a leading malignancy of the gastrointestinal tumour, posing a substantial risk to human health and substantially diminishing quality of life and overall survival.^125^, ^126^, ^127^ Studies have confirmed that METTL1‐mediated m7G methylation regulates the let‐7e miRNA/HMGA2 axis to suppress CC progression. It is particularly important to uncover the molecular and cellular mechanisms that underline CC progression, as doing so will provide valuable understanding into disease mechanisms and opportunities for novel therapeutic development.

BC research has increasingly focused on the m7G methylation pathway, revealing its critical involvement in RNA metabolism, gene expression regulation and malignant progression. In certain molecular contexts, METTL1‐mediated tRNA m7G induces translational dysregulation, leading to cell‐cycle arrest and inhibiting tumourigenesis. Furthermore, integrative models combining m7G regulators with tumour‐microenvironment features have been proposed as potential prognostic and therapeutic biomarker signatures in breast cancer.^66^, ^67^ m7G regulation also intersects with miRNA machinery, with aberrant expression of the m7G‐related gene modulating BC cell invasion by regulating LASP1 and let‐7a levels. Collectively, these results underscore the context‐dependent nature of m7G regulation in breast cancer.

In OSCC, METTL1/WDR4‐driven tRNA m7G modification enhances the selective translation of growth and EMT‐related transcripts, driving tumour progression.^69^ Additionally, METTL1 catalyses m7G modification on mRNA, promoting mRNA stability, increasing NEK1 expression and facilitating tumour cell proliferation.^70^ Consistent with these observations, METTL1‐driven tRNA m7G also reprogrammes tumour metabolism, contributing to acquired resistance to anlotinib.^77^ Genetic or pharmacologic attenuation of METTL1 restores drug sensitivity both in vitro and in vivo, implicating the m7G‐translation‐metabolism axis as a potential therapeutic vulnerability. These findings further confirm METTL1's role as a critical node in OSCC, with both tRNA‐dependent translational control and mRNA‐centred regulation, including metabolic reprogramming, contributing to the disease phenotype.

BCa is the ninth most common malignancy and 30% of them belong to muscle‐invasive bladder cancer. Its pathogenesis is characterized by rapid progression, early metastasis and poor prognosis. Despite radical surgery and adjuvant chemotherapy, more than half of muscle‐invasive bladder cancer patients die from cancer metastasis.^128^, ^129^, ^130^ METTL1 expression levels are closely associated with BCa progression. METTL1 knockdown inhibits cancer progression in xenograft mouse models and zebrafish cancer models. METTL1‐mediated m7G tRNA methylation promotes BCa development through the EGFR pathway.^48^

AML has a poor prognosis and low survival rate. For more than four decades, cytotoxic chemotherapy intensified with cytarabine + anthracycline (7 + 3) has been the traditional approach for the treatment of AML. With whole‐genome level sequencing, clinicians are using molecular analysis of AML to risk stratify patients and guide treatment.^131^, ^132^, ^133^, ^134^ eIF4E selectively enhances mRNA export and translation by binding to the m7G cap, leading to leukemic progression.^73^

Malignant gliomas are clinically characterized by high mortality, poor prognosis and elevated recurrence rate. Despite limited studies on the molecular mechanisms underlying glioma, emerging evidence has highlighted the critical role of m7G methylation in glioma prognosis.^135^, ^136^, ^137^, ^138^ METTL1 is strongly associated with glioma progression. Studies have indicated that METTL1‐mediated m7G methylation activates the MAPK pathway, promoting glioma growth and proliferation.^37^ More recent functional studies have shown that METTL1 further drives glioma progression by catalysing m7G modification and stabilizing PGK1 mRNA, a glycolysis‐related enzyme, thereby enhancing glycolytic flux, accelerating cancer cells growth and survival in vivo.^71^ Additionally, a comprehensive epitranscriptomic analysis of glioma further contextualizes m7G regulators, including METTL1/WDR4 and cap‐dependent readers, within key signalling networks, highlighting their clinical relevance in glioma progression.^72^

### 1.11 Target specificity of m7G in cancer

As mechanistic understanding deepens, loss‐of‐function studies of METTL1/WDR4 in cells and animal models further validate target effects and specificity. In HCC and ICC, knockout of METTL1/WDR4 lowers global tRNA m7G levels, suppresses codon‐biased translation of cell‐cycle and survival mRNAs, and attenuates tumour growth in vitro and vivo. Crucially, re‐expression of catalytically competent METTL1 restores both the m7G mark and malignant phenotypes, whereas catalytically impaired mutants do not, demonstrating the target specificity.^47^, ^48^, ^130^, ^139^ In OSCC, knockout of METTL1 or knockdown of WDR4 in OSCC cells reduced proliferation, migration and survival by lowering tRNA m7G levels, which inhibited the translation of oncogenic transcripts. Orthotopic transplantation models showed that both METTL1 knockout and WDR4 knockdown significantly hindered tumour growth and lymph node metastasis.^69^ METTL1 knockdown also significantly reduces NEK1 expression and decreases the abundance of m7G modification at the 5′ untranslated region (UTR) of NEK1 mRNA, which in turn destabilizes the transcript.^70^ In glioma, METTL1 depletion impairs the m7G modification of PGK1 mRNA, leading to a decrease in PGK1 expression, which suppresses both glioma cell glycolysis in vitro. This reduction in PGK1 expression results in slower tumour growth in vivo, underscoring the critical position of METTL1 mediated m7G in glioma. These findings further confirm the importance of m7G regulation in tumour metabolism and cellular proliferation.^71^ By contrast, cancer suppression in LC/CC/BC, METTL1 depletion compromises pri‐miRNA maturation (the let‐7 and miR‐149‐3p programmes) and decreased METTL1 disrupts translation of GADD45A and RB1, ultimately resulting in cancer progression, whereas METTL1 re‐expression restores the phenotype, indicating that the direction of effect is context‐locked to the operative RNA regulon.^9^, ^66^, ^74^ These studies confirm the complex regulatory actions of m7G in cancer and demonstrate the specificity of target roles. This opens significant opportunities for the future process of targeted strategies, with the potential to design more effective and precise treatments that specifically target m7G‐related pathways.

### 1.12 Clinical applications of m7G RNA methylation modifications

The core machinery of m7G RNA methylation shows considerable prospective as a promising biomarker for disease diagnosis and outcome prediction across multiple cancer types. In LC, METTL1/WDR4‐mediated m7G tRNA modification is markedly upregulated and closely associated with poor clinical outcomes, reflecting its oncogenic role. Similarly, elevated METTL1‐mediated m7G tRNA modification has been detected in HCC tissues, reinforcing the importance of aberrant m7G methylation in tumour progression. Multiple studies have revealed that aberrant upregulation of the methyltransferases METTL1 and WDR4 is significantly associated with progressive tumour stage and aggressive vascular infiltration, and poor clinical outcomes in HCC patients, highlighting their potential as prognostic biomarkers and therapeutic targets.^49^ In the same way, recent research highlights the role of METTL1 overexpression in enhancing resistance to radiotherapy, primarily by promoting the efficiency of non‐homologous end‐joining‐mediated DNA double‐strand break repair.^140^ Furthermore, Expression levels of METTL1 and WDR4 were found to be highly elevated in lenvatinib‐resistant HCC cells, suggesting that aberrant activation of the METTL1/WDR4‐mediated m7G pathway may underlie therapeutic resistance and that these enzymes could serve as biomarkers to predict lenvatinib efficacy.^76^ Therefore, given its pronounced correlation with cancer stages and patient outcomes, the METTL1/WDR4 complex may serve as a promising molecular biomarker and potentially therapeutic stratification of HCC.

Beyond its diagnostic and prognostic value, m7G RNA methylation modification also offers a promising avenue for therapeutic intervention in a wide range of cancers, providing opportunities to disrupt oncogenic translation and tumour progression. m7G modification regulates miRNA function through the control of let‐7 family members, and targeting METTL1 to promote the maturation of miRNA processing may open new therapeutic avenues. m7G tRNA modification mediated by METTL1 results in selective translation of mRNAs that play an important role in ICC. Experimental studies in preclinical mouse models of ICC have revealed that pharmacological or genetic inhibition of METTL1 enhances the therapeutic potency of anti‐PD‐1 immunotherapy, suggesting a potential synergistic therapeutic strategy.^141^ In ICC, METTL1 catalyses m7G tRNA modification, which regulates the codon‐dependent translational efficiency of CXCL8 and CXCL5 mRNAs. This process enhances the production of pro‐tumourigenic chemokines that contribute to immune modulation and cancer progression. Modulation of this selective translation mechanism by targeting METTL1 may be key to the development of drugs for ICC. Beyond HCC and ICC, OSCC exhibits METTL1‐driven tRNA m7G‐dependent metabolic reprogramming that promotes acquired resistance to anlotinib, whereas genetic or pharmacologic attenuation of METTL1 restores sensitivity, nominating an m7G‐translation‐metabolism axis as a tractable target.^77^ The METTL1‐m7G‐EGFR/EFEMP1 axis plays a key role in driving BCa progression. This mechanism not only elucidates the molecular basis of METTL1‐mediated oncogenic signalling but also gives a promising therapeutic target for BCa treatments. METTL1 enhances the chemosensitivity of HeLa cells to 5‐FU.^75^ In CC, METTL1 overexpression amplifies the cytotoxic effects of high‐dose cisplatin treatment by modulating the S100A4/p53 signalling pathway. This interaction enhances apoptosis and contributes to increased chemosensitivity.^74^ This chemoresistance mechanism provides a new targeting strategy for CC treatment in clinical settings.

In addition, eIF4E expression levels are elevated in a variety of cancers, and it can also serve as an important therapeutic target. For example,^142^ the promyelocytic leukaemia protein PML regulates its function by altering eIF4E activity. Ribavirin, a broad‐spectrum antiviral compound, exerts its anti‐tumour effects by competing with eIF4E for binding to the m7G cap. This competitive inhibition disrupts cap‐dependent translation, thereby suppressing oncogenic protein synthesis and offering therapeutic benefit in leukaemia. Therefore, targeting the eIF4E complex through competitive m7G cap inhibitors is also essential for anti‐tumour drug development.

Notwithstanding these opportunities, clinical translation requires a tempered view. Interventions along the m7G/cap axis carry off‐target risks that may perturb essential translation programmes in normal tissues.^143^ Achieving stable, cell type selective in vivo delivery of modulators for RNA‐modifying enzymes or cap‐interacting factors remains unresolved despite progress with nucleic acid and small molecule platforms. The clinical evidence base is still early: there are no registered trials directly targeting METTL1/WDR4. eIF4E‐directed strategies (LY2275796) have far shown pharmacodynamic engagement but limited efficacy in phase I,^144^ and first‐generation METTL1 inhibitors remain micromolar and preclinical, underscoring the gap between concept and clinic.^145^ Taken together, METTL1/WDR4 are promising components of an m7G biomarker axis, but routine clinical use will require standardized, orthogonally validated assays, lineage‐specific thresholds and prospective multi‐central validation.

### 1.13 Technical limitations and translational directions of m7G

Despite rapid progress, important gaps remain in the m7G field. Mechanistically, the context determinants that toggle METTL1/WDR4 outputs between oncogenic, codon‐biased selective translation and miRNA‐centred tumour suppression are incompletely resolved at the patient level. Integrated, multi‐omic datasets that jointly profile tRNA m7G stoichiometry, codon usage, mRNA structure, miRNA processing efficiency and eIF4E paralog balance across cohorts are scarce.^146^ Methodologically, reliable detection and quantification of m7G still face constraints: antibody‐based enrichment can suffer from cross‐reactivity and batch variability, undermining specificity; chemistry or RT‐signature based sequencing improves site resolution but typically requires substantial input, is sensitive to RNA integrity or secondary structure, and may introduce sequence‐context biases limitations that are particularly acute for tRNA.^21^, ^22^, ^24^ By contrast, LC–MS/MS affords sensitive global quantification yet lacks nucleotide resolution and is vulnerable to matrix and pre‐analytical effects.^147^ Complementing these constraints, emerging live‐cell platforms such as CRISPR RiPCA now enable quantitative measurement of eIF4E–m7GpppX capped mRNA interactions and on‐target activity of cap‐binding inhibitors in cells, providing an orthogonal pharmacodynamic readout and screening tool that bridges detection and translation.^148^ Translationally, off‐target risks remain salient because interventions on the m7G/cap axis can perturb essential translation programmes in normal tissues,^143^ while in vivo delivery of modulators for RNA‐modifying enzymes or cap‐interacting factors is not yet reliably stable, cell‐type‐selective and endosomally escaping. In addition, the clinical evidence base is nascent, with no registered trials directly targeting METTL1/WDR4, only pharmacodynamic signals from an eIF4E antisense approach in phase I,^144^ and first‐generation METTL1 inhibitors remaining micromolar and preclinical.^145^ Addressing these gaps will require, at minimum, assay standardization with orthogonal validation, combining site‐specific sequencing with LC–MS/MS and rigorously calibrated spike‐in controls—to produce reproducible, cross‐study m7G readouts suitable for clinical deployment. In parallel, prospectively designed, multi‐central cohorts with predefined cut‐offs and external validation are needed to establish analytic validity, clinical validity and ultimately clinical utility. Equally important, the field should advance potent, selective chemical probes or degraders against METTL1/WDR4 and cap readers, coupled with clinically feasible delivery solutions, to enable mechanism‐based intervention. A complementary priority is to test biomarker‐guided combination strategies, for example, pairing m7G translation axis modulators with targeted agents or immunotherapies, within rational trial designs. These efforts could move m7G from a compelling biological axis to clinically actionable diagnostics and therapeutics, improving patient stratification, overcoming resistance and expanding treatment options.

## 2 CONCLUSION

RNA modifications are integral to cancer biology. Among them, m7G acts through METTL1/WDR4 and eIF4E to shape translation and RNA processing across coding and non‐coding transcripts, yielding oncogenic or tumour‐suppressive outputs in a context‐dependent manner. This pattern is consistent with broader epitranscriptomic frameworks that link RNA methylation to cancer mechanisms and clinical opportunities.^10^, ^11^, ^12^ Although current studies establish the central role of m7G in tumourigenesis, a balanced appraisal must acknowledge translational barriers: interventions on the m7G/cap axis carry off‐target risks that may perturb essential translation programmes in normal tissues,^143^ and achieving stable, cell‐type‐selective in vivo delivery of modulators for RNA‐modifying enzymes or cap‐interacting factors remains unresolved. Moreover, the clinical evidence base is still nascent—no registered trials directly target METTL1/WDR4, eIF4E‐directed strategies have so far shown only pharmacodynamic engagement in phase I, and first‐generation METTL1 inhibitors remain micromolar and preclinical.^144^, ^145^ Taken together, while the m7G axis offers promising avenues for biomarkers and therapy, realizing clinical benefit will require potency‐selective agents, validated and orthogonal assays, effective delivery solutions and rigorously designed prospective trials with rational patient stratification. It is anticipated that continued investigation into m7G methylation modification will facilitate the discovery and development of more specific therapeutic agents and precision treatment strategies targeting this pathway in cancer, and such strategies undoubtedly have a long way to go from theory to practice.

## AUTHOR CONTRIBUTIONS

PengYuan Dang and ZhenQiang Sun provided direction and guidance throughout the preparation of this manuscript. PengYuan Dang wrote and edited the manuscript. KaiBo Li reviewed and made significant revisions to the manuscript. All authors read and approved the final manuscript. All authors contributed to the article and approved the submitted version.

## CONFLICT OF INTEREST STATEMENT

The authors declare no conflicts of interest.

## ETHICS STATEMENT

Not applicable as this is a review article. No human or animal subjects were involved in this study by the authors.
